# *N*′-[(*E*)-2-Hy­droxy-3,5-diiodo­benzyl­idene]pyridine-3-carbohydrazide

**DOI:** 10.1107/S160053681103176X

**Published:** 2011-08-11

**Authors:** A. Thirugnanasundar, J. Suresh, A. Ramu, G. RajaGopal

**Affiliations:** a Department of Chemistry, Velalar College of Engineering and Technology, Erode 638009, India; b Department of Inorganic Chemistry, Madurai Kamaraj University, Madurai 625 021, India; c Department of Physics, The Madura College, Madurai 625 021, India; d Department of Chemistry, Government Arts College, Melur 625 106, India

## Abstract

In the title compound, C_13_H_9_I_2_N_3_O_2_, the dihedral angle between the two aromatic rings is 10.5 (2)°. The mol­ecule displays a *trans* configuration with respect to the C=N bond. An intra­molecular O—H⋯N hydrogen bond occurs. The crystal packing is stabilized by N—H⋯O and C—H⋯O hydrogen bonds.

## Related literature

For the biological activity of isoniazid derivatives, see: Janin (2007 ▶); Kahwa *et al.* (1986 ▶); Chen *et al.* (1997 ▶); Ren *et al.* (2002 ▶). For a related structure, see: Zhi & Wang (2010 ▶). For hydrogen-bond motifs, see: Bernstein *et al.* (1995 ▶).
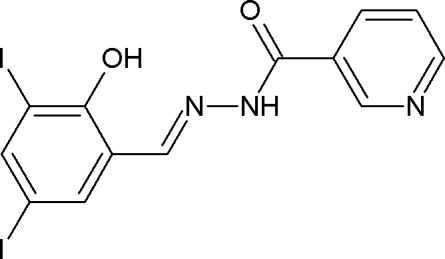


## Experimental

### 

#### Crystal data

C_13_H_9_I_2_N_3_O_2_
*M* *_r_* = 493.03
Monoclinic, 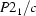

*a* = 17.4800 (5) Å
*b* = 8.5710 (4) Å
*c* = 9.8650 (3) Å
β = 90.451 (4)°
*V* = 1477.94 (9) Å^3^
*Z* = 4
Mo *K*α radiation
μ = 4.26 mm^−1^
*T* = 293 K
0.25 × 0.22 × 0.19 mm

#### Data collection

Bruker APEXII CCD area-detector diffractometer
Absorption correction: multi-scan (*SADABS*; Sheldrick, 2003 ▶) *T*_min_ = 0.359, *T*_max_ = 0.445
19735 measured reflections
4866 independent reflections
3640 reflections with *I* > 2σ(*I*)
*R*_int_ = 0.024

#### Refinement

*R*[*F* ^2^ > 2σ(*F* ^2^)] = 0.034
*wR*(*F* ^2^) = 0.106
*S* = 0.99
4866 reflections
183 parameters
H-atom parameters constrained
Δρ_max_ = 1.68 e Å^−3^
Δρ_min_ = −1.17 e Å^−3^

### 

Data collection: *APEX2* (Bruker, 2004 ▶); cell refinement: *SAINT* (Bruker, 2004 ▶); data reduction: *SAINT*; program(s) used to solve structure: *SHELXS97* (Sheldrick, 2008 ▶); program(s) used to refine structure: *SHELXL97* (Sheldrick, 2008 ▶); molecular graphics: *ORTEP-3* (Farrugia, 1997 ▶); software used to prepare material for publication: *SHELXL97* and *PLATON* (Spek, 2009 ▶).

## Supplementary Material

Crystal structure: contains datablock(s) global, I. DOI: 10.1107/S160053681103176X/bt5587sup1.cif

Structure factors: contains datablock(s) I. DOI: 10.1107/S160053681103176X/bt5587Isup2.hkl

Supplementary material file. DOI: 10.1107/S160053681103176X/bt5587Isup3.cml

Additional supplementary materials:  crystallographic information; 3D view; checkCIF report

## Figures and Tables

**Table 1:** Hydrogen-bond geometry (Å, °)

*D*—H⋯*A*	*D*—H	H⋯*A*	*D*⋯*A*	*D*—H⋯*A*
O1—H1*A*⋯N1	0.82	1.88	2.599 (4)	146
N2—H2⋯O2^i^	0.86	2.13	2.962 (4)	163
C7—H7⋯O2^i^	0.93	2.59	3.367 (4)	142

Symmetry code: (i) 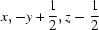.

**Fig. 1.**
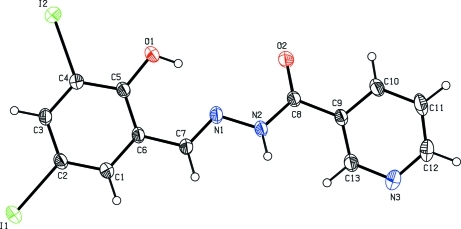
The structure of showing the atom-numbering scheme. The displacement ellipsoids are drawn at the 30% probability level. The disordered atoms are omitted for clarity.

**Fig. 2.**
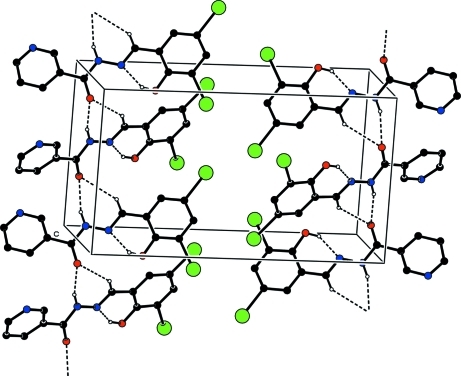
A diagram showing the formation of R21(6) bifurcated ring and S(6) motif of molecules of the title compound through N–H···O, C–H···O and O–H···N hydrogen bonding.

## supplementary crystallographic information

### Comment

In the search of new compounds, isoniazid derivatives have been found to possess potential tuberculostatic activity (Janin, 2007). Schiff bases have attracted much attention because of their biological activity (Kahwa *et al.*, 1986). Some of the compounds have been found to have excellent pharmacological and antibacterial activity (Chen *et al.*,1997; Ren *et al.*, 2002). Against this background, and in order to obtain detailed information on molecular conformations in the solid state, an X-ray study of the title compound was carried out.

X-Ray analysis confirms the molecular structure and atom connectivity as illustrated in Fig. 1. The pyridine ring is essentially planar with maximum deviations of 0.012 (5) Å, for atom C12. The molecular structure of the title compound displays a *trans* configuration with respect to the C═N and C—N bonds. The dihedral angle between the benzene and the pyridine rings is 10.5 (2)°. The atoms I1, I2 and O1 are deviated by -0.008 (1), -0.054 (1) and -0.050 (3) Å from the leastsquares plane of the benzene ring. All the bond lengths are within normal ranges and comparable to those in other similar compound (Zhi & Wang, 2010).

The intramolecular O1–H1A···N1 hydrogen bond completes a six-membered ring, which generates an S(6) motif (Bernstein *et al.*, 1995). Atoms N2 and C7 act as donors to form bifurcated hydrogen bonds with atom O2 as an acceptor, results in the formation of *R*^2^_1_(6) bifurcated ring. In addition to van der Waals interaction, the crystal packing is stabilized N–H···O, O–H···N and C–H···O hydrogen bonds and further stabilized by weak intermolecular C2–I1···*Cg*2^ii^ interaction involving the centroid of the benzene ring (*Cg*2 is the centroid of the benzene ring; ii= *x*,1/2 - *y*,-1/2 + *z*).

### Experimental

An methanol solution (20 ml) and 3,5-diiodosalicylaldehyde (10 mmol) was magnetically stirred in a round bottom flask after getting clear solution followed by addition of Nicotinic hydrazide (10 mmol).The reaction mixture was refluxed for two hours and upon cooled to room temperature yellow crystalline solid was precipitated from the mixture it was recrystaliced with Dimethylformamide single yellow crystal were obtained washed with methanol and air dried. Single crystals suitable for X-ray diffraction were obtained by slow evaporation of a solution of the title compound in acetone at room temperature.

### Refinement

All H atoms were fixed geometrically and allowed to ride on their parent atoms, with C—H distances fixed in the range 0.93–0.97 Å, N—H = 0.86Å and O—H = 0.82Å. *U*_iso_(H) were set to  1.5*U*_eq_(C) for methyl H and 1.2*U*_eq_(C, N, O) for other H atoms.

### Crystal data

**Table:**

C_13_H_9_I_2_N_3_O_2_	*F*(000) = 920
*M**_r_* = 493.03	*D*_x_ = 2.216 Mg m^−^^3^
Monoclinic, *P*2_1_/*c*	Mo *K*α radiation, λ = 0.71073 Å
Hall symbol: -P 2ybc	Cell parameters from 4867 reflections
*a* = 17.4800 (5) Å	θ = 1.2–31.6°
*b* = 8.5710 (4) Å	µ = 4.26 mm^−^^1^
*c* = 9.8650 (3) Å	*T* = 293 K
β = 90.451 (4)°	Block, white
*V* = 1477.94 (9) Å^3^	0.25 × 0.22 × 0.19 mm
*Z* = 4	

### Data collection

**Table:**

Bruker APEXII CCD area-detector diffractometer	4866 independent reflections
Radiation source: fine-focus sealed tube	3640 reflections with *I* > 2σ(*I*)
graphite	*R*_int_ = 0.024
ω and φ scans	θ_max_ = 31.6°, θ_min_ = 2.3°
Absorption correction: multi-scan (*SADABS*; Sheldrick, 2003)	*h* = −25→25
*T*_min_ = 0.359, *T*_max_ = 0.445	*k* = −12→12
19735 measured reflections	*l* = −14→12

### Refinement

**Table:**

Refinement on *F*^2^	Secondary atom site location: difference Fourier map
Least-squares matrix: full	Hydrogen site location: inferred from neighbouring sites
*R*[*F*^2^ > 2σ(*F*^2^)] = 0.034	H-atom parameters constrained
*wR*(*F*^2^) = 0.106	*w* = 1/[σ^2^(*F*_o_^2^) + (0.0525*P*)^2^ + 1.9568*P*] where *P* = (*F*_o_^2^ + 2*F*_c_^2^)/3
*S* = 0.99	(Δ/σ)_max_ = 0.001
4866 reflections	Δρ_max_ = 1.68 e Å^−^^3^
183 parameters	Δρ_min_ = −1.17 e Å^−^^3^
0 restraints	Extinction correction: *SHELXL97* (Sheldrick, 2008), Fc^*^=kFc[1+0.001xFc^2^λ^3^/sin(2θ)]^-1/4^
Primary atom site location: structure-invariant direct methods	Extinction coefficient: 0.0015 (2)

### Special details

**Table:**

Geometry. All e.s.d.'s (except the e.s.d. in the dihedral angle between two l.s. planes) are estimated using the full covariance matrix. The cell e.s.d.'s are taken into account individually in the estimation of e.s.d.'s in distances, angles and torsion angles; correlations between e.s.d.'s in cell parameters are only used when they are defined by crystal symmetry. An approximate (isotropic) treatment of cell e.s.d.'s is used for estimating e.s.d.'s involving l.s. planes.
Refinement. Refinement of *F*^2^ against ALL reflections. The weighted *R*-factor *wR* and goodness of fit *S* are based on *F*^2^, conventional *R*-factors *R* are based on *F*, with *F* set to zero for negative *F*^2^. The threshold expression of *F*^2^ > σ(*F*^2^) is used only for calculating *R*-factors(gt) *etc*. and is not relevant to the choice of reflections for refinement. *R*-factors based on *F*^2^ are statistically about twice as large as those based on *F*, and *R*- factors based on ALL data will be even larger.

### Fractional atomic coordinates and isotropic or equivalent isotropic displacement parameters (Å^2^)

**Table:**

	*x*	*y*	*z*	*U*_iso_*/*U*_eq_	
C1	0.28906 (18)	0.3439 (4)	0.7602 (3)	0.0383 (7)	
H1	0.2713	0.2819	0.6892	0.046*	
C2	0.36332 (17)	0.3990 (4)	0.7591 (3)	0.0364 (6)	
C3	0.39014 (17)	0.4933 (4)	0.8622 (3)	0.0367 (6)	
H3	0.4397	0.5328	0.8598	0.044*	
C4	0.34258 (17)	0.5285 (4)	0.9691 (3)	0.0371 (6)	
C5	0.26788 (16)	0.4720 (4)	0.9750 (3)	0.0347 (6)	
C6	0.24076 (16)	0.3806 (4)	0.8670 (3)	0.0343 (6)	
C7	0.16298 (17)	0.3211 (4)	0.8627 (3)	0.0381 (7)	
H7	0.1459	0.2645	0.7880	0.046*	
C8	0.00141 (15)	0.2795 (4)	1.0619 (3)	0.0347 (6)	
C9	−0.07420 (15)	0.2011 (4)	1.0435 (3)	0.0338 (6)	
C10	−0.13285 (18)	0.2377 (5)	1.1314 (4)	0.0491 (9)	
H10	−0.1259	0.3125	1.1987	0.059*	
C11	−0.2021 (2)	0.1607 (5)	1.1169 (4)	0.0559 (10)	
H11	−0.2425	0.1819	1.1748	0.067*	
C12	−0.2097 (2)	0.0528 (5)	1.0153 (4)	0.0548 (9)	
H12	−0.2569	0.0043	1.0046	0.066*	
C13	−0.08758 (17)	0.0874 (4)	0.9470 (3)	0.0419 (7)	
H13	−0.0477	0.0612	0.8894	0.050*	
N1	0.11794 (15)	0.3469 (3)	0.9622 (3)	0.0394 (6)	
N2	0.04524 (14)	0.2851 (4)	0.9506 (3)	0.0385 (6)	
H2	0.0284	0.2511	0.8740	0.046*	
N3	−0.15404 (18)	0.0128 (4)	0.9311 (4)	0.0546 (8)	
O1	0.22488 (13)	0.5069 (3)	1.0828 (2)	0.0458 (6)	
H1A	0.1816	0.4722	1.0714	0.069*	
O2	0.02167 (13)	0.3328 (3)	1.1712 (2)	0.0481 (6)	
I1	0.434960 (14)	0.34479 (3)	0.59728 (3)	0.05286 (10)	
I2	0.385470 (15)	0.66245 (4)	1.12850 (3)	0.06771 (12)	

### Atomic displacement parameters (Å^2^)

**Table:**

	*U*^11^	*U*^22^	*U*^33^	*U*^12^	*U*^13^	*U*^23^
C1	0.0354 (14)	0.0453 (18)	0.0344 (15)	−0.0064 (12)	0.0072 (11)	−0.0015 (12)
C2	0.0329 (13)	0.0427 (17)	0.0336 (14)	−0.0020 (12)	0.0085 (11)	0.0027 (12)
C3	0.0273 (12)	0.0402 (17)	0.0425 (16)	−0.0042 (11)	0.0051 (11)	0.0005 (13)
C4	0.0312 (13)	0.0416 (17)	0.0384 (15)	−0.0028 (11)	0.0036 (11)	−0.0044 (12)
C5	0.0301 (12)	0.0424 (17)	0.0318 (14)	−0.0012 (11)	0.0050 (10)	0.0014 (12)
C6	0.0268 (12)	0.0439 (17)	0.0324 (14)	−0.0055 (11)	0.0042 (10)	0.0026 (12)
C7	0.0304 (13)	0.0504 (19)	0.0336 (14)	−0.0077 (12)	0.0035 (11)	−0.0011 (13)
C8	0.0278 (12)	0.0457 (17)	0.0306 (13)	−0.0019 (11)	0.0026 (10)	0.0022 (12)
C9	0.0260 (12)	0.0463 (16)	0.0291 (13)	−0.0010 (11)	0.0023 (10)	0.0072 (12)
C10	0.0318 (14)	0.075 (3)	0.0405 (17)	−0.0020 (15)	0.0078 (12)	−0.0049 (17)
C11	0.0305 (15)	0.083 (3)	0.054 (2)	−0.0024 (16)	0.0129 (14)	0.0105 (19)
C12	0.0334 (16)	0.065 (2)	0.066 (2)	−0.0117 (15)	0.0036 (15)	0.010 (2)
C13	0.0314 (14)	0.053 (2)	0.0412 (17)	−0.0026 (13)	0.0049 (12)	0.0016 (14)
N1	0.0289 (11)	0.0539 (17)	0.0354 (13)	−0.0087 (10)	0.0033 (10)	0.0044 (11)
N2	0.0276 (11)	0.0598 (17)	0.0280 (11)	−0.0081 (11)	0.0039 (9)	0.0021 (11)
N3	0.0393 (15)	0.060 (2)	0.065 (2)	−0.0134 (13)	0.0004 (14)	−0.0036 (16)
O1	0.0350 (11)	0.0663 (17)	0.0363 (12)	−0.0061 (10)	0.0104 (9)	−0.0084 (11)
O2	0.0360 (11)	0.0756 (19)	0.0326 (12)	−0.0084 (11)	0.0010 (9)	−0.0064 (11)
I1	0.04408 (14)	0.06674 (19)	0.04806 (15)	−0.00641 (10)	0.01933 (10)	−0.00998 (11)
I2	0.04370 (14)	0.0942 (3)	0.06543 (19)	−0.02088 (13)	0.00969 (12)	−0.03975 (15)

### Geometric parameters (Å, °)

**Table:**

C1—C2	1.382 (4)	C8—N2	1.344 (4)
C1—C6	1.391 (4)	C8—C9	1.492 (4)
C1—H1	0.9300	C9—C13	1.381 (5)
C2—C3	1.378 (4)	C9—C10	1.384 (4)
C2—I1	2.089 (3)	C10—C11	1.385 (5)
C3—C4	1.381 (4)	C10—H10	0.9300
C3—H3	0.9300	C11—C12	1.369 (6)
C4—C5	1.394 (4)	C11—H11	0.9300
C4—I2	2.082 (3)	C12—N3	1.329 (5)
C5—O1	1.341 (4)	C12—H12	0.9300
C5—C6	1.402 (4)	C13—N3	1.334 (4)
C6—C7	1.452 (4)	C13—H13	0.9300
C7—N1	1.282 (4)	N1—N2	1.381 (3)
C7—H7	0.9300	N2—H2	0.8600
C8—O2	1.221 (4)	O1—H1A	0.8200
			
C2—C1—C6	120.3 (3)	N2—C8—C9	115.3 (3)
C2—C1—H1	119.9	C13—C9—C10	118.0 (3)
C6—C1—H1	119.9	C13—C9—C8	123.1 (3)
C1—C2—C3	120.6 (3)	C10—C9—C8	118.8 (3)
C1—C2—I1	119.9 (2)	C11—C10—C9	118.6 (4)
C3—C2—I1	119.5 (2)	C11—C10—H10	120.7
C4—C3—C2	119.2 (3)	C9—C10—H10	120.7
C4—C3—H3	120.4	C12—C11—C10	118.5 (3)
C2—C3—H3	120.4	C12—C11—H11	120.7
C3—C4—C5	121.7 (3)	C10—C11—H11	120.7
C3—C4—I2	118.8 (2)	N3—C12—C11	124.3 (3)
C5—C4—I2	119.5 (2)	N3—C12—H12	117.8
O1—C5—C4	119.1 (3)	C11—C12—H12	117.8
O1—C5—C6	122.6 (3)	N3—C13—C9	124.2 (3)
C4—C5—C6	118.3 (3)	N3—C13—H13	117.9
C1—C6—C5	119.9 (3)	C9—C13—H13	117.9
C1—C6—C7	118.2 (3)	C7—N1—N2	116.2 (3)
C5—C6—C7	121.9 (3)	C8—N2—N1	118.5 (3)
N1—C7—C6	119.8 (3)	C8—N2—H2	120.8
N1—C7—H7	120.1	N1—N2—H2	120.8
C6—C7—H7	120.1	C12—N3—C13	116.4 (3)
O2—C8—N2	123.0 (3)	C5—O1—H1A	109.5
O2—C8—C9	121.8 (3)		
			
C6—C1—C2—C3	−1.2 (5)	C5—C6—C7—N1	−2.7 (5)
C6—C1—C2—I1	−179.4 (2)	O2—C8—C9—C13	−152.5 (3)
C1—C2—C3—C4	1.9 (5)	N2—C8—C9—C13	26.8 (5)
I1—C2—C3—C4	−179.8 (2)	O2—C8—C9—C10	24.1 (5)
C2—C3—C4—C5	−0.5 (5)	N2—C8—C9—C10	−156.6 (3)
C2—C3—C4—I2	177.2 (2)	C13—C9—C10—C11	−1.0 (5)
C3—C4—C5—O1	178.5 (3)	C8—C9—C10—C11	−177.8 (3)
I2—C4—C5—O1	0.8 (4)	C9—C10—C11—C12	−0.6 (6)
C3—C4—C5—C6	−1.6 (5)	C10—C11—C12—N3	2.1 (7)
I2—C4—C5—C6	−179.3 (2)	C10—C9—C13—N3	1.5 (5)
C2—C1—C6—C5	−1.0 (5)	C8—C9—C13—N3	178.1 (3)
C2—C1—C6—C7	179.4 (3)	C6—C7—N1—N2	−179.4 (3)
O1—C5—C6—C1	−177.7 (3)	O2—C8—N2—N1	3.4 (5)
C4—C5—C6—C1	2.3 (5)	C9—C8—N2—N1	−175.9 (3)
O1—C5—C6—C7	1.9 (5)	C7—N1—N2—C8	166.1 (3)
C4—C5—C6—C7	−178.0 (3)	C11—C12—N3—C13	−1.7 (6)
C1—C6—C7—N1	177.0 (3)	C9—C13—N3—C12	−0.2 (6)

### Hydrogen-bond geometry (Å, °)

**Table:**

*D*—H···*A*	*D*—H	H···*A*	*D*···*A*	*D*—H···*A*
O1—H1A···N1	0.82	1.88	2.599 (4)	146
N2—H2···O2^i^	0.86	2.13	2.962 (4)	163.
C7—H7···O2^i^	0.93	2.59	3.367 (4)	142.

Symmetry codes: (i) *x*, −*y*+1/2, *z*−1/2.

## References

[bb1] Bernstein, J., Davis, R. E., Shimoni, L. & Chang, N.-L. (1995). *Angew. Chem. Int. Ed. Engl.* **34**, 1555–1573.

[bb2] Bruker (2004). *APEX2* and *SAINT* Bruker AXS Inc., Madison, Wisconsin, USA.

[bb3] Chen, H. Q., Hall, S., Zheng, B. & Rhodes, J. (1997). *BioDrugs*, **7**, 217–231. 10.2165/00063030-199707030-00005 18031095

[bb4] Farrugia, L. J. (1997). *J. Appl. Cryst.* **30**, 565.

[bb5] Janin, Y. L. (2007). *Bioorg. Med. Chem.* **15**, 2479–2513. 10.1016/j.bmc.2007.01.030 17291770

[bb6] Kahwa, I. A., Selbin, J., Hsieh, T. C.-Y. & Laine, R. A. (1986). *Inorg. Chim. Acta*, **118**, 179–185.

[bb7] Ren, S., Wang, R., Komatsu, K., Bonaz-Krause, P., Zyrianov, Y., McKenna, C. E., Csipke, C., Tokes, Z. A. & Lien, E. J. (2002). *J. Med. Chem.* **45**, 410–419. 10.1021/jm010252q 11784145

[bb8] Sheldrick, G. M. (2003). *SADABS* University of Göttingen, Germany.

[bb9] Sheldrick, G. M. (2008). *Acta Cryst.* A**64**, 112–122. 10.1107/S0108767307043930 18156677

[bb10] Spek, A. L. (2009). *Acta Cryst.* D**65**, 148–155. 10.1107/S090744490804362X PMC2631630 19171970

[bb11] Zhi, F. & Wang, R. (2010). *Acta Cryst.* E**66**, o892. 10.1107/S1600536810010020 PMC2984034 21580709

